# An Attempt to Simplify the Treatment of Sexual Diseases

**Published:** 1831

**Authors:** 


### AN ATTEMPT TO SIMPLIFY THE TREATMENT OF SEXUAL DISEASES. By *James Thorn*, Member of the Royal College of Surgeons. 8vo. pp. 240. Highley, London, 1831.

IF there be wisdom in the multitude of books upon a subject, then in syphilis are we wise. To mention the mere names of the authors on this most prolific topic, were to engage in a task as arduous as that of Homer, when he invoked a hundred tongues and a voice of brass to aid him in his somewhat lengthy catalogue of the Grecian ships. One would imagine that every debatable point had been debated, that every practical question had been settled, that theorists could theorize no longer, that routine practitioners had made up their minds how to cure or to kill. Fortunately for physic and its professors that millenium is not arrived; fortunately there yet remain doubts which will never be solved, books to be written, men to be infected, and money to be made. So long as men differ in the constitution of their minds as well as in the conformation of their bodies, so long will there be disputable points in medicine, and so long will syphilis be one of them. It must be admitted that this investigation is surrounded with peculiar difficulties, with difficulties, we say, inherent in itself. Independent of the natural complexities of a disease which assumes many forms, is much influenced by constitutional peculiarities, and modified by remedial measures, the inquirer is met at almost every step by a disposition to deceive on the part of the patient, unequalled under other circumstances. How fallacious is the syphilitic history of a case, how little is narration of bye-gone events to be depended on. In short, we repeat that it requires no prophet to foretel, that the disputes upon syphilis which have heretofore reigned are not likely to be very materially curtailed.

Mr. Thorn, the author of the volume whose title is registered above is an industrious and indefatigable young surgeon. Many of our readers may probably remember that this gentleman directed the attention of the professional public some three years ago, to the employment of an extract of copaiba in the treatment of gonorrhœa. As the author makes no pretension to important discoveries or brilliant hypotheses, starts few new facts and fewer speculations, we shall not subject his work to the ordeal of a methodic critique. An extract or two will afford a fair sample of the whole, and these, though not absolutely taken at random, are certainly not selected as the very best passages that might be brought forward. Here is a description of inflammation of the glans penis.

"Habitual uncleanness of the penis, the continued contact of the mucous matter and of urine under the prepuce, intercourse too frequent, or with a woman whose vagina is very narrow, and whose mucous membrane is inflamed, are the occasional causes of this.

The glans is, in this case, redder, shining, swelled, and more tender than usual, and the patient feels an itching and burning heat. Sometimes we observe a decided ulceration. At other times the integuments are entire; but, on comparing them, we perceive some puriform matter dropping from an indefinite number of small points. The glans as well as the whole penis is swelled, kept either partly or wholly erected, and the least touch, or even the motion in walking, gives severe pain.

If, in this case, the inflammation spreads to the *meatus urinarius*, pain is produced by the passage of urine.

If it fixes on the *corona glandis*, the secretion, first suppressed, afterwards takes place abundantly; there is also matter exhaled by the surface of the glans; and, from their union, a white, yellowish, or muddy fluid is produced at the surface of the prepuce, which is at first thick and afterwards serous.

Sometimes the skin of the glans dries up and falls off in flakes.

In this stage the inflammation is but trifling, and gives way quickly. It nevertheless, generally extends to the prepuce, and may produce inflammation of the testicles and of the glands of the groin.

In the course of the urethral inflammation, the surface of the glans becomes sleek, semi-transparent, livid and red, particularly under the *meatus urinarius*. Sometimes it seems excoriated, is very tender, and emits a yellow fœtid matter, at first thick, then liquid.

The swelling of the glans may proceed so far, that the patient loses the power of drawing back the prepuce, when it is long and narrow, or may even be unable to bring it back over the glans, after having made it pass the corona glandis, which constitutes phymosis in the first case and paraphymosis in the second, both already described as arising from another and more usual cause.

*Treatment of Inflammation of the Glans.* Superficial inflammation, without ulceration, in the acute state, requires a soothing regimen, with mild aperients, frequent fomentation of the parts, and, above all, the application of two or three leeches when severe.

The chronic state first requires emollient applications, and then a solution of the acetate of lead or zinc.

In both cases, cooling drinks at first, then slightly aromatic watery decoction, are used; and every thing ought to be avoided, which might otherwise irritate the urinary and digestive organs." 65.

*Inflammation of the Genital Organs in Women.* Our author observes that the inflammatory symptoms vary according to the situation, the mode of sensibility, and the functions of the part affected. There are, therefore, four principal varieties, which may exist independently of each other, or may be connected.

"In the first case the inflammation occupies the vagina only, the canal becomes swelled, the patient feels in its whole extent a particular sensation of constriction, and the orifice is sometimes so swelled as to prevent the introduction of the finger. The mucous membrane is at first dry, or but very little moistened, but the pain and heat soon increase in intensity, and a discharge takes place.

This state of the disease is less troublesome, but not more common, than others.

The inflammation begins at the entrance of the vagina, its more sensible part, and it communicates by degrees to the extent of this canal in such a manner, as to occupy a considerable portion of the mucous membrane.

In the second variety, the irritation soon extends itself to the urethra, to the clitoris, to the extremity of the vulva, and even to the pubis. Generally the swelling is very painful; the woman feels a sensation similar to that which is produced by the presence of a foreign body, having a continual tendency to extend beyond the vulva, and the urine, in passing, produces heat, tension, and disagreeable stiffness, which soon becomes a severe pain.

All these symptoms increase progressively as the inflammation goes on. Touching the parts, and the contact of the dress, becomes insupportable; frequent erections of the clitoris take place, which add very much to the torments of the patient; and the secretion, which is trifling at the beginning, now becomes very abundant.

The urethra itself is the seat of the inflammation in the third variety. The symptoms are then the same as in men, at least as far as concerns the influence of the disease in passing the urine, which occasions at first a tickling, accompanied with heat; and, in the greatest number of cases, the sensibility of the canal increases in a few days, the desire of making water becomes frequent, its passage occasions great pain, and an abundant discharge takes place from the urethra.

At last, in the fourth variety, the nymphæ, the labiæ, the posterior commissure of the vulva, and the fossa navicularis, are the parts affected with the inflammation.

There is a difficulty attending this disease in the female, that of deciding when she has the disease, and when she is cured. When the woman has an intention to deceive, it is far from easy to come to a decision on these points. In the commencement there will usually be itching and titillation of the orifice of the urethra, some swelling of the labiæ and nymphæ, and the part will often appear more red and injected.

Foreign substances introduced and left in the vagina have often caused intense and sometimes chronic inflammation. Inquiry, therefore, should always be made with due reserve, as to the possibility of a cause of this kind, whenever there is the least probability of it.

Frequently inflammation of the vagina is consequent upon that of the womb. It is important to attend to this point.

All running from the vagina, indeed, depends upon the injury done to it or to the womb, in consequence of coition or some other cause. All running then of this kind offers various problems to solve.

Whenever a woman, therefore, has an intent of concealing the cause of the running which she experiences, we are never certain on what circumstance it depends; and nothing is more doubtful than the origin of discharge when we would discover it by questions.

The diseased parts must therefore be examined by feeling and inspection, instead of depending upon vain theories or false answers. What is material, is not so much to know whether the malady proceeds from coition, as its extent and intensity, and whether the womb is affected by it, and if it is not the sole source of the matter which runs from the vagina.

Even when the woman acknowledges that it proceeds from coition we must nevertheless make sure that the womb does not partake of it, particularly when it is of long standing." 132.

The following is the summary of the treatment to be employed.

" These inflammations get well often when left to themselves, provided the patients adhere to a proper diet, and keep the affected parts from the action of causes capable of increasing their state of irritation.

Art may, however, accelerate the cure, soothe the violence of the symptoms, and remedy those which often shew themselves when the proper rules have been violated. With this view we must follow the same course as in inflammation of the urethra in man.

The woman ought, from the commencement, to avoid every thing which can increase the inflammation, and determine the flow of blood to the affected organs, such as exercise on horseback, walking, the act of coition, voluptuous or excited feelings, wine or spirits, high-seasoned food, &c.

Rest and the recumbent position are equally necessary in the early period of the complaint.

Care, in respect to cleanliness, is still more necessary in women than in men.

The antiphlogistic method is proper, especially where the irritation is propagated to the urinary organs, when the patient experiences acute pain, fever, and difficulty of making water.

Bleeding from the foot, leeches to the vulva, warm baths, general or partial vapour bath, injections of warm water or a mucilaginous liquid, emollient washings, mild laxatives, as cassia pulp and castor oil, repose in bed, acidulated drinks, strict diet, or at least a very soothing and unirritating diet, are the means which diminish, in a few days, the inflammatory symptoms.

In addition to these means the extract of copaiba must be given in doses of from ten to fifteen grains three times a day, and the *Decoct. Tormentill.* used as an injection very frequently.

It is useful to anoint the perineum, and the internal part of the thighs, with white cerate, to guard against the contact of the puriform excretion which would irritate them; but if recourse is had early to the use of Tormentilla decoction as a lotion, and injection of the internal parts, these accidents seldom occur.

If the discharge continues in spite of these remedies, sea-bathing will be found to contribute to the completion of the cure." 138.

### ULCERS OF THE GENITAL ORGANS.

We cannot afford space nor time to follow Mr. Thorn seriatim through this portion of his work, its better part. It is chiefly occupied with a sort of running commentary on Messrs. Evans and Carmichael, and though judicious, we find but little originality. We shall content ourselves with a gleaning here and there. In the following case, our author conceives that the irritation and ulceration produced by pediculi were followed by secondary symptoms. We give the case as we find it, but we cannot forbear from remarking that, for very obvious reasons, it is far from conclusive.

"Mr. S. ætatis 23, consulted me in January 1829, complaining of considerable irritation, and uneasiness in the pubis, which, on examination, was clearly seen to arise from the existence of pediculi in that part, and from the account he gave, it was probable these animals had been there for several weeks.

The skin of the pubis in many parts was covered with scales, and bled from those points where the pediculi had penetrated.

He had never contracted a gonorrhœa, or suffered from any form of ulceration of the genital organs previous to this time. I directed him to apply the following lotion.

Hydr. Oxymuriat. grs. ix.

Sp. Vin. Rectific. ℥j.

Aqua Distill. f℥iij. M. ft. Lotio."

A week afterwards, this gentleman called on me again, when it appeared that the pediculi were destroyed, but from several points of ulceration running into each other, two tolerably large sores were formed upon the pubis. The hair was removed from the part, and the ulcer dressed with *Cerat. Merc. Præp.* The following morning the edges of the ulcers were slightly touched with *Argent. Nitras*, in consequence of the indolent character they assumed, and under this treatment, in about ten days, they healed. Six weeks afterwards I saw this gentleman, when he had an eruption over the whole of his body of a papular character, which had every appearance of that form which is usually denominated syphilitic, and the sequela of certain ulcers of the generative organs. He had considerable pain and uneasiness in the throat, the tonsils were exceedingly swollen, and ulceration was extending rapidly towards the soft palate.

Having seen the patient in the commencement, I was very desirous of watching the train of symptoms following the ulcers, being fully convinced that the primary ulcers of the pubis were produced solely by the pediculi having been suffered to exist for some time.

The strictest attention was paid to the regulation of the digestive functions and general health, he was ordered a gargle of solution of Chloruret of Soda to the throat, the use of the warm bath daily, and to take the Decoct. Sarsa. Comp.; under this plan all the symptoms got well in about three weeks." 157.

With one more extract on the effects of iodine frictions in the treatment of buboes we must conclude.

"One of the most useful applications is iodine, as it is active, has the advantage of being easily managed, and acts quite locally.

We may apply the ointment of the hydriodate of potash, but the tincture of iodine is much more useful. It is best used by itself in frictions. When incorporated in lard, or suspended in an oily menstruum, it is seldom efficacious.

The doses employed are of one or two drachms per diem, according to the size of the tumour, its duration, and excitability of the patient. The frictions ought to be repeated frequently in the course of the day, and continued each time five or six minutes.

The effect which it produces is to make the skin yellow, to dry up the epidermis, which falls off in large scales, to occasion pricking, shooting, and also slight pains, to increase at length the organic action of the parts, and consequently to favour the resolution of the adjacent tumours.

A poultice must be applied immediately after the friction.

When the frictions are carefully made, we usually observe a diminution of the swelling at the end of four or five days; and often at the end of from eight to ten days the cure is completed.

It often happens, however, that the tumours, in consequence of their magnitude or long duration, require much more time, and sometimes prove refractory.

In these cases the internal use of iodine may be combined advantageously with frictions, in doses of fifteen, twenty, or thirty drops of the Tincture, night and morning, in any convenient vehicle. The excitement of the intestines which it occasions has an alterative effect, and assists the resolution.

When iodine causes acute pain its use may be suspended for a time, and again had recourse to in a diminished dose.

If the skin becomes red, painful, and inflamed, leeches must be used.

In this manner the excitation is kept up in a manner suitable to resolution, and preventing the formation of pus.

Iodine has been used with great advantage for removing the hardness and swelling which remain after the cure of ulcerated buboes." 229.

Here, as we have already said, we must conclude. Having placed several extracts before our readers, we need scarcely pronounce a formal sentence. Whilst we consider the work not undeserving of approbation, particularly as being the production of an industrious and meritorious young man, we cannot conceal from the author the fact, that he has yet much to learn before he can become an accomplished writer. The style is indifferent; the composition too generally careless; not seldom incorrect. The errors of the press are multifarious, and on more than one occasion we have noticed sins against the ordinary rules of grammar. For such faults as these we hold that authors are entitled to some censure, as a little attention would correct them. We hope, on some future opportunity, to meet Mr. T. again in print; and we trust that the hints which we have given in kindness will not be thrown away.

